# Essential Oils from *Origanum vulgare* subsp. *virens* (Hoffmanns. & Link) Ietsw. Grown in Portugal: Chemical Diversity and Relevance of Chemical Descriptors

**DOI:** 10.3390/plants12030621

**Published:** 2023-01-31

**Authors:** Alexandra M. Machado, Violeta Lopes, Ana M. Barata, Orlanda Póvoa, Noémia Farinha, A. Cristina Figueiredo

**Affiliations:** 1 Centro de Estudos do Ambiente e do Mar (CESAM Lisboa), Faculdade de Ciências da Universidade de Lisboa (FCUL), Biotecnologia Vegetal, DBV, C2, Campo Grande, 1749-016 Lisboa, Portugal; 2 Banco Português de Germoplasma Vegetal (BPGV), Instituto Nacional de Investigação Agrária e Veterinária, Quinta de S. José, S. Pedro de Merelim, 4700-859 Braga, Portugal; 3 VALORIZA—Centro de Investigação para a Valorização de Recursos Endógenos, Instituto Politécnico de Portalegre, Praça do Município 11, 7300-110 Portalegre, Portugal; 4 Instituto Politécnico de Portalegre, Praça do Município 11, 7300-110 Portalegre, Portugal

**Keywords:** chemical descriptors, chemotypes, medicinal and aromatic plants, essential oils, oregano

## Abstract

*Origanum vulgare* L. is a well-known aromatic and medicinal plant, whose essential oil (EO) has recognised flavouring and medicinal properties. In this study, *Origanum vulgare* subsp. *virens* (Hoffmanns. & Link) Ietsw. EOs, isolated from accessions grown in experimental fields, were evaluated. The plant material was grown from rooted cuttings or nutlets (fruits), originally collected in 20 regions in mainland Portugal and harvesting for EO isolation was performed in two years. EOs were isolated by hydrodistillation and analysed by gas chromatography and gas chromatography–mass spectrometry, for EO quantification and identification, respectively. EO yields ranged from <0.05–3.3% for rooted cuttings, with oregano samples obtained in Portalegre and Alandroal, respectively. Ninety-one compounds were identified, mainly grouped in oxygen-containing monoterpenes and monoterpene hydrocarbons. EO agglomerative cluster analysis evidenced two main clusters, with the first subdivided into four subclusters. From the obtained data, the putative *O. vulgare* subsp. *virens* chemotypes are carvacrol, thymol and linalool, with γ-terpinene, *p*-cymene, *cis*- and *trans*-β-ocimene also contributing as these EOs chemical descriptors. The comparison between the present data and a survey of the existing literature on Portuguese *O. vulgare* reinforces the major variability of this species’ EOs and emphasises the importance of avoiding wild collections to obtain a defined chemical type of crop production of market relevance.

## 1. Introduction

*Origanum vulgare* L. (Lamiaceae), commonly known as oregano, is considered the most variable species of the genus, being found throughout the Mediterranean region, and also in most parts of the Euro-Siberian and Irano-Turanian regions [1,2,3]. However, the *Origanum* genus is mainly distributed around the Mediterranean region, with 38 described species [3]. Since Ietswaart’s revision [3], eight more species have been described in *Origanum*, and the total count can be higher if subspecies and hybrids are included [3,4,5]. *O. vulgare* has been collected since ancient times, with appreciated flavour and recognised medicinal properties, used in traditional dishes and used to relieve several complaints of the respiratory tract, such as convulsive coughs, colds and asthma, and also painful menstruation, rheumatoid arthritis, urinary tract or digestive disorders [6,7].

Ietswaart’s [3] taxonomic revision of the genus *Origanum* distinguished six subspecies of *O. vulgare* L. according to morphological characters, specifically, *gracile* (K.Kock) Ietswaart, *glandulosum* (Desfontaines) Ietswaart, *hirtum* (Link) A. Terracc., *vulgare* L., *virens* (Hoffmanns. & Link) Ietsw. and *viridulum* (Martrin-Donos) Nyman. These subspecies were differentiated based on indumentum variances, number of oil glands and size/colour of bracts and flowers [2]. In addition to morphological differences, a great diversity in EO yield and composition was also observed, according to the geographical origin and distinct environmental conditions [8,9,10,11].

In Portugal, there is only one wild species, *Origanum vulgare* L., comprising two subspecies, *O. vulgare* subsp. *virens* (Hoffmanns. & Link) Ietsw. and *O. vulgare* subsp. *vulgare* L. [12,13]. The subsp. *virens*, occasionally called Moroccan origanum, is the common oregano, naturally widespread throughout the several regions, particularly on field edges, well-drained soils, uncultivated lands or shrublands. Subsp. *vulgare* is mostly distributed in the northwest of the country [12,14,15].

*O. vulgare* subsp. *virens* is traditionally used both in Portugal (mainland, Azores and Madeira Islands) [16,17] and Spain for the treatment of respiratory and skin infections and as a stomachic and antispasmodic [18]. This species was considered as one of the most popular Lamiaceae used as a food additive in the Trás-os-Montes province of Portugal [19].

The main compounds identified in studies with *O. vulgare* and *O. vulgare* subsp. *virens* EOs are monocyclic monoterpenes, mostly the phenol-like oxygen-containing monoterpenes, carvacrol and thymol. Other chemically related compounds also present in different amounts include γ-terpinene, α-terpinene, *p*-cymene, *p*-cymenene, carvacrol and thymol methyl ethers and acetates, terpinen-4-ol, *p*-cymen-8-ol and *p*-cymen-7-ol [4,20,21,22,23].

Among the several subspecies of *O. vulgare* L., different EO yields are obtained. According to De Mastro et al. [24] and Lukas et al. [2], oreganos with EO yields ≥2% show carvacrol and/or thymol as well as their biosynthetic precursors γ-terpinene and *p*-cymene as main EO compounds. The predominance of phenolic monoterpenes gives the pungent characteristic oregano flavour with higher commercial interest. In contrast, in plants with a lower EO yield the main constituents comprise acyclic compounds, mostly linalool, β-ocimene or β-myrcene, and bicyclic compounds such as sabinene, *cis*- and/or *trans*-sabinene hydrate. Borneol and bornyl acetate can also be found, and also high amounts of sesquiterpenes such as β-caryophyllene, germacrene D, bicyclogermacrene, α- and γ-muurolene and β-caryophyllene oxide [2,24,25,26,27,28].

Given the main identified monoterpene compounds, several chemotypes have been recognised in oregano EOs, such as α-terpineol, and 1,8-cineole chemotypes, terpinene-4-ol and β-myrcene chemotypes or citronellol-rich chemotypes [24,29,30,31]. However, there is a great diversity of oregano essential oil characteristics when considering the total identified monoterpenes, which makes it difficult to describe the diversity of EOs present in spontaneous *O. vulgare*. Moreover, the EOs obtained from northern Mediterranean countries, including Portugal, and of Central and Northern Europe, showed low yield, which contributes to the scarcity of studies [2,24].

Both the Portuguese Pharmacopoeia IX [32] and the European Pharmacopoeia 10 [33] include a monograph about oregano, which comprises the dried leaves and flowers of two species, *O. onites* L. and *O. vulgare* L. subsp. *hirtum* (Link) Ietsw., alone or mixed. This monograph also addresses the EO quantification, referring to at least 60% carvacrol and thymol. The ISO 13171:2016 [34] only considers the essential oil of *O. vulgare* L. subsp. *hirtum* (Link) Ietsw, with carvacrol (60–80%) having dominance.

These data highlight the lack of information on the dominant subspecies in Portugal, *O. vulgare* subsp. *virens*, both at the morphological and essential oil composition variability level. More comprehensive knowledge on *O. vulgare* chemical diversity in Portugal is required considering the valorisation and protection of the national biodiversity. These studies are also important since they provide information about the country’s plant resources, in order to complement existing morphological descriptors [35,36] with chemical traits. 

The goal of this work was to evaluate Portuguese *O. vulgare* subsp. *virens* EO variability, and to provide putative EO chemical descriptors to complement the existing morpho-agronomic characters, such as plant’s height and flowering patterns, among others. To accomplish this goal, 38 *O. vulgare* subsp. *virens* EOs were isolated from accessions of 20 different geographical origins, established in experimental fields, either from rooted cuttings or by nutlets (fruits). In addition, the obtained data were compared with a survey of the previous existing literature on Portuguese *O. vulgare* EOs.

## 2. Results

**Figure 1:**
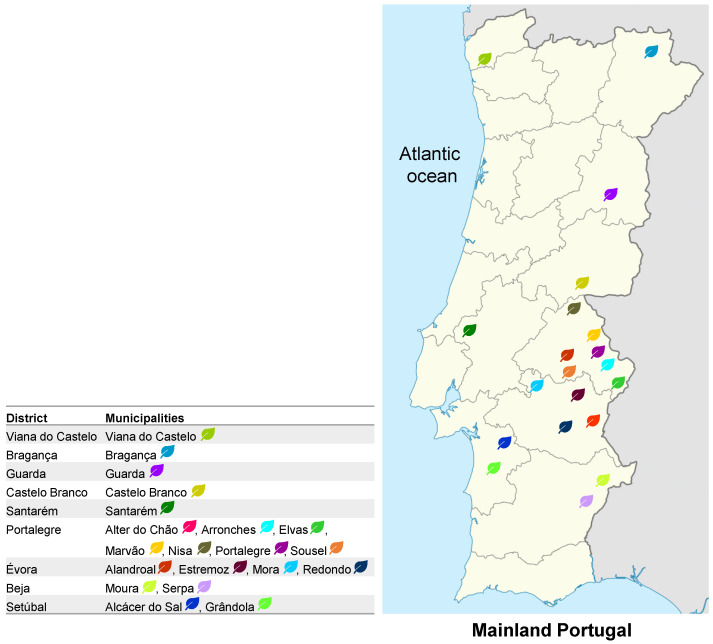
Mainland Portugal geographical origin of the studied oregano samples (n = 38).

In total, 38 *Origanum vulgare* subsp. *virens* EOs were isolated from the accessions grown in the experimental fields of Escola Superior Agrária de Elvas/Instituto Politécnico de Portalegre (ESAE/IPP) and Banco Português de Germoplasma Vegetal (BPGV)/INIAV, shown in Table 1. Oregano accessions were obtained either from rooted cuttings or from nutlets, originating from 20 different regions in mainland Portugal, shown in Table 1 and Figure 1.

### 2.1. EO Yield, Composition and Cluster Analysis

Essential oil yields ranged between <0.05 and 3.3% (*v*/*w*) for oregano rooted cutting-obtained accessions grown in Elvas, and <0.1–0.6% for nutlet-obtained accessions grown in Braga, shown in Table 1 and Figure 1. The EOs showed different colours according to plants’ original provenance and propagation material type, shown in Supplementary Material Figure S1.

The relative amounts of all the identified compounds are listed in Supplementary Material Table S1a,b. In total, ninety-one volatile compounds were identified in the analysed samples, accounting for 90–100% of the total composition. Oxygen-containing monoterpenes (10–91%) and monoterpene hydrocarbons (3–76%) were the main grouped EO components. 

Irrespective of the propagation material type used, linalool (traces, t-84%), carvacrol (t-52%) and thymol (t-50%) were the dominant compounds. γ-Terpinene (t-40%) and *p*-cymene (t-27%), followed by β-caryophyllene (17–19%), α-terpineol (t-18%), *cis*-β-ocimene (11–13%) and *trans*-β-ocimene (9–13%) were also detected in relatively high percentages.

**Figure 2:**
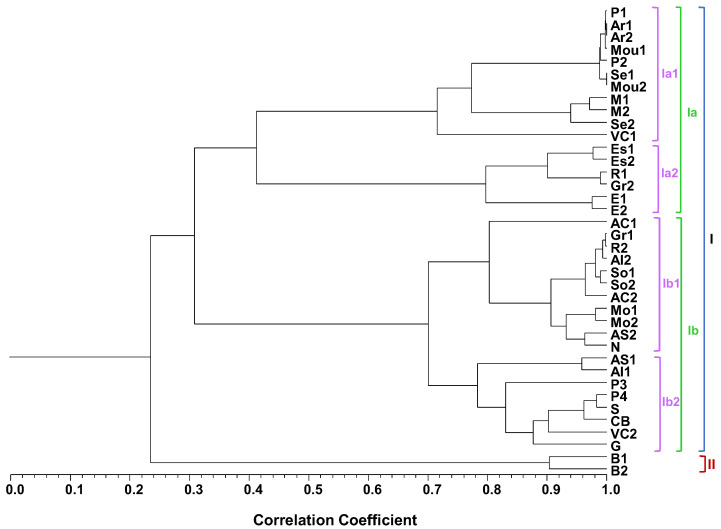
Dendrogram obtained by cluster analysis of the percentage composition of the essential oils isolated by hydrodistillation from the 38 samples, based on correlation, and using the unweighted pair group method with arithmetic average (UPGMA). For harvest site codes, *vide* Table 1.

Hierarchical clustering was used to evaluate the oregano EO chemical composition correlation, shown in Figure 2 and Table 2. Cluster analysis evidenced a dendrogram with two main clusters, cluster I and II, with cluster I subdivided into four subclusters, shown in Figure 2.

Despite the low correlation (Scorr < 0.25) of clusters I and II, oregano accessions from the same propagation material type and growing site (Elvas, Table 1) were assigned to the same cluster and subcluster, highlighting their volatiles’ similarity. The observed differences were mostly due to the variable proportions of some of the dominant compounds.

Oregano EOs isolated from samples obtained from rooted cuttings were distributed among the three subclusters in clusters I, Ia1, Ia2 and Ib1, with the exception of samples AS1 and Al1 positioned in subcluster Ib2. Linalool (20–84%), shown in Table 2, was the dominant compound in the highly correlated samples of subcluster Ia1 (Scorr < 0.75), shown in Figure 2, derived from plants originally from Portalegre, Arronches, Moura, Serpa and Marvão, shown in Table 1. The sample obtained from nutlets from Viana do Castelo was also included in this subcluster.

Subcluster Ia2 grouped highly correlated rooted cutting samples (Scorr < 0.82), shown in Figure 2, collected in Estremoz, Redondo, Grândola and Elvas, with carvacrol as the dominant compound (23–52%), shown in Table 2. Samples with thymol as the dominant compound (21–50%), shown in Table 2, also showed high correlation (Scorr < 0.82). Included in subcluster Ib1, these samples comprised plant material from Alter do Chão, Grândola, Redondo, Alandroal, Sousel, Mora, Alcácer do Sal and Nisa.

**Table 1:** Data on *Origanum vulgare* subsp. *virens* accessions studied, their codes, harvest site, type of propagation material, experimental field of growth, plantation and harvest dates and EO yields.

Accession Code	BPGV Accession #	Harvest Site	Propagation Material Type	Experimental Field	Plantation Date (Month/Year)	Harvest Date (Month/Year)	EO Yield (%, *v*/*w*)
Al1	BPGV28151	Alandroal	rooted cuttings	ESAE/IPP	04/21	10/21	2.99
Al2						07/22	3.33
AS1	BPGV27704	Alcácer do Sal	rooted cuttings	ESAE/IPP	04/21	10/21	2.88
AS2						07/22	3.26
AC1	BPGV28152	Alter do Chão	rooted cuttings	ESAE/IPP	04/21	10/21	2.86
AC2						07/22	2.37
Ar1	BPGV27690	Arronches	rooted cuttings	ESAE/IPP	04/21	10/21	1.98
Ar2						07/22	2.82
B1	BPGV11267	Bragança	nutlets	BPGV	05/21	06/22	<0.05
B2	BPGV11280						<0.05
CB	BPGV16427	Castelo Branco	nutlets	BPGV	05/21	06/22	0.19
E1	BPGV19647	Elvas	rooted cuttings	ESAE/IPP	04/21	10/21	2.85
E2						07/22	2.89
Es1	BPGV19646	Estremoz	rooted cuttings	ESAE/IPP	04/21	10/21	2.38
Es2						07/22	2.32
Gr1	BPGV27691	Grândola	rooted cuttings	ESAE/IPP	04/21	10/21	1.77
Gr2						07/22	2.49
G	BPGV12102	Guarda	nutlets	BPGV	05/21	06/22	0.39
M1	BPGV27684	Marvão	rooted cuttings	ESAE/IPP	04/21	10/21	1.44
M2						07/22	1.73
Mo1	BPGV27692	Mora	rooted cuttings	ESAE/IPP	04/21	10/21	1.76
Mo2						07/22	1.56
Mou1	BPGV28153	Moura	rooted cuttings	ESAE/IPP	04/21	10/21	2.58
Mou2						07/22	2.15
N	BPGV28154	Nisa	rooted cuttings	ESAE/IPP	11/21	07/22	2.44
P1	BPGV27708	Portalegre	rooted cuttings	ESAE/IPP	04/21	10/21	<0.05
P2						07/22	1.48
P3	BPGV10423		nutlets	BPGV	05/21	06/22	0.39
P4							0.56
R1	BPGV27686	Redondo	rooted cuttings	ESAE/IPP	04/21	10/21	2.43
R2						07/22	2.20
S	BPGV11408	Santarém	nutlets	BPGV	05/21	06/22	0.28
Se1	BPGV27709	Serpa	rooted cuttings	ESAE/IPP	04/21	10/21	2.92
Se2						07/22	2.39
So1	BPGV27705	Sousel	rooted cuttings	ESAE/IPP	04/21	10/21	2.10
So2						07/22	1.94
VC1	BPGV16272	Viana do Castelo	nutlets	BPGV	05/21	06/22	<0.05
VC2	BPGV16286						0.11

ESAE/IPP: Escola Superior Agrária de Elvas/Instituto Politécnico de Portalegre. BPGV: Banco Português de Germoplasma Vegetal.

Subcluster Ib2 mainly comprised highly correlated (Scorr < 0.80) EOs obtained from oregano nutlets from Portalegre, Santarém, Castelo Branco, Viana do Castelo and Guarda, all grown in Braga (BPGV), shown in Table 1. These EOs evidenced γ-terpinene (21–40%) and *p*-cymene (5–27%) as the main compounds, shown in Table 2. This cluster also included two EOs obtained from rooted cuttings in 2021, AS1 and Al1, from Alcácer do Sal and Alandroal, grown in Elvas, shown in Table 1.

Cluster II grouped the highly correlated (Scorr < 0.92) B1 and B2 EOs, obtained from oregano nutlets from Bragança and grown in Braga (BPGV), shown in Table 1. β-Caryophyllene (17–19%), *cis*- and *trans*-β-ocimene (11–13%, 9–13%, respectively) and γ-terpinene (4–13%) were these EOs’ dominant compounds, shown in Table 2.

Even if, in general, EOs obtained in different years from samples with the same provenance grouped in a common cluster, there were some exceptions. R1 EO exhibited carvacrol as the dominant compound in 2021 (25%) and only 0.4% thymol, whereas in R2 EO, thymol dominated in 2022 (40%), with carvacrol only reaching 1%. EOs obtained from Grândola oregano samples also displayed differences, since in 2021 the dominant compound was thymol (39% thymol, 3% carvacrol) and carvacrol in the following year (25% carvacrol, 1% thymol). EOs obtained from oregano samples collected in Alandroal showed *p*-cymene (27%), γ-terpinene (21%) and thymol (15%) as the main compounds in 2021. Nevertheless, in 2022 thymol was the dominant compound (41%), followed by *p*-cymene (18%) and γ-terpinene (14%). Similar results were observed for EOs obtained from oregano samples collected in Alcácer do Sal, which presented γ-terpinene (32%), *p*-cymene (25%) and thymol (11%) as major compounds in 2021. However, in 2022 both thymol and γ-terpinene were dominant (27%), followed by *p*-cymene (23%).

### 2.2. Comparative Evaluation with Published Data on Portuguese O. vulgare EOs

The phytochemical constituents of *O. vulgare* grown in mainland Portugal and Madeira Island, namely their EOs, have been previously studied, shown in Table 3, confirming a high chemical polymorphism, and the existence of several chemotypes [6,14,18,21,22,27,37,38,39,40].

Although both the isolation and analytical procedures were similar, the plant parts, the physiological stage, the plant status (fresh or dry) and the geographical origin were quite diverse, or not reported, in addition to the use of collective wild samples. Despite this variability, a cluster analysis of the EOs’ reported data evidenced two main clusters with very low correlation (Scorr < 0.10), with the second subdivided into three subclusters, also with a very low correlation (Scorr < 0.20), shown in Table 4 and Supplementary Material Figure S2.

Cluster I was dominated by α-terpineol (9–66%), whereas this compound was either not reported or <26% in all cluster II samples, shown in Table 4.

Cluster II gathered the reported data that shared compounds present in all samples, and subclusters, in similar ranges, such as *p*-cymene (3–14%), or less common compounds, such as β-fenchyl alcohol (13%). The subclusters of cluster II were mainly differentiated by the high ranges of thymol (19–58%) in subcluster IIa, γ-terpinene (15–49%) in subcluster IIb and carvacrol (14–68%) in subcluster IIc.

**Table 2:** Minimum and maximum percentage range of EOs’ main components (≥3% in at least one sample) isolated from the different *Origanum vulgare* subsp. *virens* accessions grouped according to cluster analysis. For samples grouped in each of the clusters and subclusters, see Figure 2. Full detailed composition is provided in SM Table S1a,b.

Components	RI	Cluster I									Cluster II
		Ia1		Ia2		Ib1		Ib2			
		Min	Max	Min	Max	Min	Max	Min	Max	Min	Max
α-Thujene	924	t	2.2	1.6	3.5	0.2	5.0	1.0	3.7	0.5	0.6
β-Myrcene	975	0.1	2.6	1.8	2.3	0.3	2.7	1.2	3.5	1.2	1.4
α-Terpinene	1002	0.1	2.5	2.3	2.8	0.3	4.9	2.3	5.8	1.3	2.7
*p*-Cymene	1003	0.2	15.1	7.5	23.1	5.3	23.6	**4.6**	**27.1**	0.8	1.8
*cis*-β-Ocimene	1017	0.8	10.2	0.5	3.7	0.5	2.8	1.5	10.2	**10.8**	**13.0**
*trans*-β-Ocimene	1027	0.1	6.5	0.1	0.5	0.2	0.6	0.3	5.8	**8.8**	**13.4**
γ-Terpinene	1035	0.4	14.5	11.4	19.5	2.4	26.8	**21.2**	**40.0**	**4.4**	**13.4**
Linalool	1074	**20.3**	**84.1**	0.3	19.9	0.2	11.0	0.2	17.7	2.5	2.7
α-Terpineol	1159	0.1	0.2	0.1	0.2	0.1	8.2	0.1	17.6	0.3	0.3
Thymol methyl ether	1210	t	3.7	t	0.4	0.1	2.1	0.5	3.8	0.1	0.5
Carvacrol methyl ether	1224	0.3	4.6	1.1	5.4	0.1	4.7	0.8	5.9	0.8	4.6
Thymol	1275	0.1	23.0	0.3	16.1	**21.2**	**50.2**	8.0	27.2	1.6	2.7
Carvacrol	1286	0.2	9.2	**23.1**	**51.5**	0.1	10.4	t	1.0	0.5	0.5
β-Caryophyllene	1414	1.1	11.5	0.8	3.5	0.4	2.0	0.8	4.4	**16.6**	**18.5**
Germacrene D	1474	0.5	7.6	0.2	1.3	0.2	1.2	0.2	3.0	7.6	8.4
Bicyclogermacrene	1487	0.3	5.5	0.1	0.9	0.1	0.7	0.2	2.5	1.4	1.7
β-Bisabolene	1500	0.3	2.3	0.6	3.2	0.8	4.7	0.6	1.2	t	t
% Identification		90.2	100.0	96.9	100.0	96.2	99.7	94.1	99.5	89.6	90.4
Grouped components											
Monoterpene hydrocarbons		3.1	42.5	30.7	47.8	10.2	63.9	48.0	75.6	41.9	45.8
Oxygen-containing monoterpenes		28.5	90.5	44.6	66.2	33.5	75.3	15.6	40.7	9.8	12.0
Sesquiterpene hydrocarbons		3.2	27.9	1.9	9.2	1.1	10.0	3.1	9.2	30.5	32.0
Oxygen-containing sesquiterpenes		0.1	1.8	0.2	0.3	t	0.6	0.1	0.6	1.5	2.2
Others		0.1	1.3	t	0.2	t	0.2	0.1	1.3	2.0	2.3

RI: In-lab calculated retention index relative to C_9_–C_16_ *n*-alkanes on the DB-1 column. Min: minimum. Max: maximum. t: traces (<0.05%). Bold: dominant compounds relevant for each cluster.

## 3. Discussion

### 3.1. EO Yield, Composition and Cluster Analysis

In the present study, oregano plants with different origin, propagation material type and developmental stages were studied regarding EO yield and chemical composition. The obtained results follow those of Nurzynska-Wierdak [8], since younger plants’ EO yield was lower than that obtained with more developed ones.

In this work, the highest EO yields (2–3%) were obtained for samples gathered in subclusters Ia2 and Ib1, with carvacrol and thymol as dominant compounds. Nevertheless, high EO yields (0.1–3%) were also obtained in linalool- and γ-terpinene-dominated EOs. In the present study, the propagation material type, rooted cuttings or nutlets, seemed to be more determinant in the EO yield, with the latter giving, in general, lower EO yields. Thus, opposite to previous reports [2,24,29], *O. vulgare* L. subsp. *virens* should not be considered a poor source of EO.

**Table 3:** Previous studies on Portuguese *Origanum vulgare* EOs.

Portugal		Aerial Parts				EO	EO Main Components Percentage (≥5%)	Code *	Ref.
Districts	HS	PS	PMS	IP	AP	Yield (%)			
Braga, Viana do Castelo, Vila Real **	Wild	V	Fresh	H	GC, GC-MS	nr	Linalool 16, δ-elemene 13, β-caryophyllene 11, α-terpineol 9, *trans*-β-ocimene 7, germacrene B 7	1998_B_V	[21]
Coimbra	Wild	F	Dried	H	GC, GC-MS	nr	α-Terpineol 66, β-caryophyllene 11, *trans*-β-ocimene 6	2012_C_F	[18]
Coimbra	Wild	V	Fresh	H	GC, GC-MS	<0.05	Carvacrol 14, *cis*-sabinene hydrate 14, γ-terpinene 10, terpinen-4-ol 8, methyl carvacrol 8, bicyclogermacrene 6, linalool 5	2013_C_V	[40]
Évora	Wild	F	Fresh	H	GC, GC-MS	1.7	Carvacrol 36, γ-terpinene 24, *p*-cymene 14, methyl carvacrol 8	2010_E_F	[27]
Évora	Wild	V	Dried	H	GC, GC-MS	0.2	γ-Terpinene 20, thymol 19, methyl thymol 13, *p*-cymene 12	2019_E_V	[22]
Faro	Local market	F	Dried	H	GC, GC-MS	1.8	Thymol 33, γ-terpinene 26, *p*-cymene 11, β-caryophyllene 5	2005_F_F	[37]
Faro	Wild	F	Dried	H	GC, GC-MS	1.8	γ-Terpinene 49, thymol 15, *p*-cymene 14, α-terpinene 5	2012_F_F	[39]
Madeira	Wild 1	nr	Dried	H	GC, GC-MS	0.9	γ-Terpinene 21, thymol 19, β-caryophyllene 9, *p*-cymene 7, methyl thymol 7	2012_M_nr_1	[14]
	Wild 2	nr	Dried	H	GC, GC-MS	1.7	Thymol 55, γ-terpinene 9, *p*-cymene 6, β-caryophyllene 5	2012_M_nr_2	[14]
	Wild 3	nr	Dried	H	GC, GC-MS	2.4	Thymol 58, γ-terpinene 10, β-caryophyllene 6, β-bisabolene 5, *p*-cymene 5	2012_M_nr_3	[14]
	Wild 4	nr	Dried	H	GC, GC-MS	0.7	Thymol 31, γ-terpinene 20, *p*-cymene 11, methyl thymol 7, β-caryophyllene 6	2012_M_nr_4	[14]
Santarém	Wild	nr	Dried	H	GC, GC-MS	nr	Carvacrol 15, β-fenchyl alcohol 13, γ-terpinene 12, 1-Methyl-3-(1-methylethyl)-benzene 7, δ-Terpineol 6	2013_S_nr	[6]
Viseu	Wild 1	F	Dried	H	GC, GC-MS	nr	γ-Terpinene 34, α-terpineol 26, *cis*-β-ocimene 6, *trans*-β-ocimene 5	2012_V_F_1	[18]
	Wild 2	F	Dried	H	GC, GC-MS	nr	Carvacrol 34, linalool 27, γ-terpinene 11, Germacrene D 5	2012_V_F_2	[18]
nr	nr	nr	nr	H	GC, GC-MS	nr	Carvacrol 68, γ-terpinene 8, *p*-cymene 7	2011_nr_nr	[38]
nr	Herbal shop 1	V	Dried	H	GC, GC-MS	1.0	α-Terpineol 16, thymol 15, γ-terpinene 15, carvacrol 10, terpinen-4-ol 9, linalool 7	2013_nr_V_1	[40]
	Herbal shop 2	V	Dried	H	GC, GC-MS	0.8	α-Terpineol 40, linalool 16, thymol 12, γ-terpinene 8, carvacrol 6	2013_nr_V_2	[40]

HS: Harvest site. PS: Plant stage. V: Vegetative. F: Flowering. PMS: Plant material status. IP: Isolation procedure. AP: Analysis procedure. Ref: Reference. H: Hydrodistillation. GC: Gas chromatography. GC-MS: Gas chromatography–mass spectrometry. nr: Not reported. * In the code, the year is followed by the first letter of the district of harvest and the second letter refers to the plant stage. ** Plant material collected at Parque Natural da Peneda Gerês that is distributed over the three districts.

**Table 4:** Minimum and maximum percentage range of EOs’ main components (≥5% in at least one sample) from previous studies on Portuguese *O. vulgare* EOs grouped according to cluster analysis. For samples grouped in each of the clusters and subclusters, *vide* SM Figure S2.

Components	Cluster I		Cluster II					
			IIa		IIb		IIc	
	Min	Max	Min	Max	Min	Max	Min	Max
α-Terpinene	t	0.7	1.4	3.4	2.0	4.7	1.2	3.7
*p*-Cymene		3.8	**4.6**	**12.2**	**3.1**	**14.1**	**3.8**	**13.8**
1-Methyl-3-(1-methylethyl)-benzene								6.8
1,8-Cineole				4.9				
*cis*-β-Ocimene	1.3	4.0	0.5	5.3	1.6	5.7	0.2	3.2
*trans*-β-Ocimene	0.8	6.8	t	6.2	0.4	4.8	0.1	1.5
γ-Terpinene	t	7.5	**8.7**	**25.9**	**14.6**	**49.1**	**7.9**	**23.5**
*cis*-Sabinene hydrate		t				t	t	14.0
Linalool	2.0	16.4	0.2	0.8	t	7.4	0.1	**27.4**
Terpinen-4-ol	t	1.8	0.1	4.0	0.4	8.5		8.1
α-Terpineol	**9.2**	**65.7**		0.2	t	**25.7**	t	1.1
δ-Terpineol								7.5
β-Fenchyl alcohol								12.8
Methyl thymol		t	1.1	13.1		0.1		0.1
Methyl carvacrol		0.1		4.4	t	0.9	0.1	8.2
Thymol		11.8	**19.4**	**58.0**	3.6	14.7		2.1
Carvacrol		5.9		6.8	0.1	10.3	**14.3**	**68.2**
δ-Elemene		12.9						0.2
β-Caryophyllene	4.0	11.1	1.8	9.1	1.7	4.3	t	3.7
Germacrene D	0.5	1.9	0.3	3.9	0.4	1.8		4.5
Germacrene B		6.6						
Bicyclogermacrene		0.9			t	1.3		5.6
β-Bisabolene	t	1.7	2.0	5.0	0.9	2.1	0.1	3.1

Min: minimum. Max: maximum. t: traces (< 0.05%). Bold: dominant compounds relevant for each cluster.

In the current study, two groups of compounds were dominant in the EOs, namely oxygen-containing monoterpenes (thymol, carvacrol, linalool) and monoterpene hydrocarbons (γ-terpinene, *p*-cymene, *cis*- and *trans*-β-ocimene). These results are in agreement with those obtained by Figuérédo et al. [41] and Gaspar and Leeke [42] for *O. vulgare* ssp. *virens* of Mediterranean origin. Figuérédo et al. [41] referred to linalool as the main compound, followed by carvacrol and the monoterpene hydrocarbons *cis*- and *trans*-β-ocimene, and Gaspar and Leeke [42] identified thymol, γ-terpinene, carvacrol, *p*-cymene and α-terpineol as main compounds. Carvacrol, thymol, γ-terpinene, *p*-cymene and linalool quantitative and qualitative differences could be related to variants of the γ-terpinene synthase gene or even its absence, as well as a predominance of cymyl and/or sabinyl pathways [43]. Sabinyl compounds, more particularly sesquiterpenes such as β-caryophyllene and germacrene D, were also identified in this study, as in other works on *O. vulgare* EOs [8,9,11,23,24,26,41,43,44].

Comparison of the obtained results with those of previous studies on Portuguese *O. vulgare* EOs [6,14,18,21,22,27,37,38,39,40] confirmed the variability in the chemical profile of oregano EOs, for individual compounds and even for their biosynthetic pathway. This variability was also reported for EO of the same species, isolated in different years and other countries [23,30,44,45,46].

EOs’ composition is mainly genetically determined although it can change with different environmental and climate conditions, seasonal harvest periods, geographic origins, plant populations, variations in cultivation conditions, extraction and quantification methods as well as with the developmental stage [9,31,47,48,49,50]. The range and diversity of the main oregano EO constituents are high, as confirmed in the present study, particularly in thymol and carvacrol content. Since these phenol-like monoterpenes impair pungent oregano flavour with high commercial potential [2,51,52], this may open a field of the improvement of oregano cultivars of great economic importance [45].

### 3.2. Origanum vulgare subsp. virens Chemotypes and Chemical Descriptors

Chemotypes can be defined as chemically distinct EO groups within a species [53]. The definition of chemotypes should not be confused with chemical differences due to different plant parts, the ratio between flowers and remaining aerial parts, using fresh or dry plant material, time between drying and EO isolation or different isolation procedures, among other factors. The type of propagation material, or even the harvest year, in addition to the material provenance can determine different main EO components, or their different ratios. For this reason, the existence of chemotypes should be determined in plant material at the same developmental stage, with similar ratios between the plant parts, with similar drying treatment if used and under similar isolation conditions.

The definition of a species chemotype is important not only from the academic point of view, but also at the commercial, industrial and medicinal level, since different chemotypes are likely to possess diverse biological properties, and thus may constitute plant resources of diverse economic relevance. This is the case, for instance, with chemical descriptors. Given the economic importance of medicinal and aromatic plants, and some of their products, such as essential oils, the importance of gathering information on countries’ plant resources’ chemical diversity to complement the existing descriptors is becoming evident.

Several works have documented the different chemotypes associated with *Origanum* spp. which confirm this species’ essential oil diversity. Sicilian oregano was found to be rich in thymol [45,54] while in Greek plants’ EOs, carvacrol was the dominant compound [51]. In Iranian oregano EO chemotypes, (*Z*)-α-bisabolene and linalyl acetate were also documented [44]. Studies with *O. vulgare* from Latvia mentioned the dominance of sesquiterpenes in the EO [23] but in southern Italy three chemotypes were identified, specifically carvacrol/thymol, thymol/α-terpineol and linalyl acetate/linalool [26]. In Turkish *O. vulgare* EO, carvacrol was the major constituent [55], whereas sabinene, *cis*-sabinene hydrate, *p*-cymene, γ-terpinene and carvacrol dominated in Austrian oregano EO [29].

These different chemotypes may have different applications, namely in the use of thymol, carvacrol and linalool in the pharmaceutical and cosmetic industries due to their antimicrobial, antioxidant, anticarcinogenesis or anti-inflammatory activities [56,57,58,59].

The variability of *O. vulgare* EO chemotypes has been attributed to different geographic locations [2,10], to the plant stage, with full flowering attaining the highest EO yield [8,14], to variability during the vegetative period [21,60] or to sexual polymorphism or genetic mechanisms related to cross pollination in specific areas [45].

Based on the obtained data in the present study, and on the survey of the previous data, carvacrol, thymol and linalool are the putative chemotypes for Portuguese *O. vulgare* subsp. *virens* EOs. Whether the presence of high levels of *p*-cymene, γ-terpinene, *cis*- *trans*-β-ocimene and β-caryophyllene reflects the existence of additional chemotypes, or metabolic and/or other types of plant variability, including the seasonality influence, requires further assessment. Nevertheless, their presence as this species’ chemical descriptors should be considered.

## 4. Materials and Methods

### 4.1. Material Sampling

*Origanum vulgare* subsp. *virens* analysed in this study was grown in the experimental fields of Escola Superior Agrária de Elvas/Instituto Politécnico de Portalegre (ESAE/IPP) and Banco Português de Germoplasma Vegetal (BPGV)/INIAV, from either oregano rooted cuttings or nutlets (fruits) obtained in different regions of mainland Portugal, as detailed in Table 1 and Figure 1. Flowering aerial parts were extracted after drying at room temperature.

### 4.2. Essential Oil Extraction

Essential oils were isolated by hydrodistillation from oregano flowering aerial parts, in a Clevenger-type apparatus according to the European Pharmacopoeia [61] for 3 h at a distillation rate of 3 mL/min. The essential oil samples were stored at −20 °C until analysis.

### 4.3. Analysis and Quantification of Compounds

Volatiles were analysed by GC for quantification and by GC-MS for component identification.

#### 4.3.1. Gas Chromatography (GC)

Essential oils were analysed using a PerkinElmer Clarus 400 gas chromatograph (PerkinElmer, Waltham, MA, USA) equipped with two flame ionisation detectors with a data handling system. Two columns of different polarities were inserted into the injector port: a DB-1 fused-silica column (100% dimethylpolysiloxane, 30 m × 0.25 mm i.d., film thickness 0.25 µm; J & W Scientific Inc., Folsom, CA, USA) and a DB-17HT fused-silica column ((50 % phenyl)-methylpolysiloxane, 30 m × 0.25 mm i.d., film thickness 0.15 µm; J & W Scientific). The oven temperature was programmed to rise from 45 to 175 °C at 3 °C/min, then to 300 °C at 15°C/min and then held isothermal for 10 min, for a total run time of 61.67 min. The split injector ratio was 1:40 and the injector and detector temperatures were 280 and 290 °C, respectively; the carrier gas was hydrogen, adjusted to a linear velocity of 30 cm/s. The percentage composition of the volatiles was computed by the normalisation method from the GC peak areas, without the use of correction factors, calculated as mean values of two injections from each sample, in accordance with ISO 7609 [62].

#### 4.3.2. Gas Chromatography–Mass Spectrometry (GC-MS)

Gas chromatography–mass spectrometry analysis was run on a PerkinElmer Clarus 690 gas chromatograph, equipped with a DB-1 fused-silica column (100% dimethylpolysiloxane, 30 m × 0.25 mm i.d., film thickness 0.25 µm; J &W Scientific), interfaced with a PerkinElmer SQ 8 T mass spectrometer (software version 6.1, PerkinElmer, Shelton, CT, USA). Injector and oven temperatures were as above; transfer line temperature, 280 °C; ion source temperature, 220 °C; carrier gas, helium, adjusted to a linear velocity of 30 cm/s; split ratio, 1:40; ionisation energy, 70 eV; scan range, 40–300 *m*/*z*; scan time, 1 s.

The identity of the components was assigned by a comparison of their retention indices (RIs), calculated in accordance with ISO 7609, relative to C_9_–C_17_ *n*-alkane (Sigma) indices and GC-MS spectra from a laboratory-made library based upon the analyses of reference essential oils, laboratory-synthesised components and commercially available standards.

### 4.4. Statistical Analysis

The percentage composition of the isolated essential oils was used to determine the relationship between the different samples by cluster analysis using the Numerical Taxonomy Multivariate Analysis System (NTSYS PC software, version 2.2, Exeter Software, Exeter University, Exeter, UK) [63]. For cluster analysis, the correlation coefficient was selected as a measure of similarity among samples and the unweighted pair group method with arithmetical averages (UPGMA) was used for cluster definition. The degree of correlation was evaluated according to Pestana and Gageiro [64] as very high [0.90, 1.00], high [0.70, 0.90[, moderate [0.40, 0.70[, low [0.20, 0.40[ and very low (<0.20).

## 5. Conclusions

There are currently six categories of descriptors that mostly gather morpho-agronomic characters (such as plant height, flowering patterns, among others), but also data from genetic markers and traditional knowledge. The chemical variability of Portuguese *O. vulgare* EOs emphasises the importance of gathering information on chemical variability to complement existing descriptors. This will contribute to the efforts to preserve the maximum genetic diversity of these natural resources, ex situ and in situ, and additionally to counteract wild plant harvest. Moreover, knowledge of the natural resources of this genus will allow a wiser use by the grower, along with contribute to avoiding the wild innate variations and help in recognising which chemotype is best suited to market demands, as well as developing cultivation methodologies, to ascertain the best propagation material for local crop production of *O. vulgare*.

Even though the results of this study showed a tendency for obtaining lower EO yield from nutlets, comparatively to that obtained from rooted cuttings, further studies are required to support these findings. This also reinforces that it will be important to consider the plant material propagation method (cuttings or nutlets) and the place of cultivation when EOs are used as chemical descriptors in germplasm banks.

The chemical profile variability of the samples studied in the present work led to the proposal of several putative chemotypes. This knowledge will be relevant to select the best fit to diverse industries, i.e., those with specific aroma as flavouring agents suitable for the food industry, and/or those with bioactive constituents appropriate to the pharmaceutical and cosmetic industries.

## Data Availability

Not applicable.

## Supplementary Materials

The following supporting information can be downloaded at: https://www.mdpi.com/article/10.3390/plants12030621/s1, Figure S1: Detail of the essential oils’ colours obtained from several oregano accessions. Portalegre (P3 and P4), Santarém (S), Viana do Castelo (VC1), Alandroal (Al2), Alcácer do Sal (AS2), Elvas (E2), Moura (Mou2). For harvest site codes, *vide* Table 1; Table S1a: Percentage composition of the EOs isolated by hydrodistillation, from *Origanum vulgare* subsp. *virens* accessions obtained from Alandroal, Alcácer do Sal, Alter do Chão, Arronches, Bragança, Castelo Branco, Elvas, Estremoz, Grândola, Guarda, in 2021 and 2022; Table S1b: Percentage composition of the EOs isolated by hydrodistillation, from *Origanum vulgare* subsp. *virens* accessions obtained from Marvão, Mora, Moura, Nisa, Portalegre, Redondo, Santarém, Serpa, Sousel, Viana do Castelo, in 2021 and 2022; Figure S2: Dendrogram obtained by cluster analysis of the reported percentage composition of the essential oils isolated by hydrodistillation from Portuguese *Origanum vulgare*, based on correlation, and using the unweighted pair group method with arithmetic average (UPGMA). In the code, the year is followed by the first letter of the district of harvest and the second letter refers to the plant stage. For harvest site codes, *vide* Table 3.
