# Supplementary material for: Essential Oils from Origanum vulgare subsp. virens (Hoffmanns. & Link) Ietsw. Grown in Portugal: Chemical Diversity and Relevance of Chemical Descriptors
Source: Plants (Basel). 2023 Jan 31;12(3):621. doi: 10.3390/plants12030621 (PMC9919071; doi:10.3390/plants12030621)
Supplement: Supplementary file 1 [file plants-12-00621-s001.zip › plants-2141300-supplementary.pdf]

plants

Article

Essential oils from *Origanum vulgare* subsp. *virens* (Hoffmanns. & Link) Ietsw. grown in Portugal. Chemical diversity and relevance of chemical descriptors

Alexandra M. Machado [1,2], Violeta Lopes [2], Ana M. Barata [2], Orlanda Póvoa [3,4], Noémia Farinha [4] and A. Cristina Figueiredo [1,*]

[1] Centro de Estudos do Ambiente e do Mar (CESAM Lisboa), Faculdade de Ciências da Universidade de Lisboa (FCUL), Biotecnologia Vegetal, DBV, C2, Campo Grande, 1749-016 Lisboa, Portugal; ialexam@gmail.com; acsf@fc.ul.pt
[2] Banco Português de Germoplasma Vegetal (BPGV), Instituto Nacional de Investigação Agrária e Veterinária, Quinta de S. José, S. Pedro de Merelim, Braga, Portugal; violeta.lopes@iniav.pt; anamaria.barata@iniav.pt
[3] VALORIZA - Centro de Investigação para a Valorização de Recursos Endógenos, Instituto Politécnico de Portalegre, Praça do Município 11, 7300-110 Portalegre, Portugal; opovoa@ipportalegre.pt
[4] Instituto Politécnico de Portalegre, Praça do Município 11, 7300-110 Portalegre, Portugal; nfarinha@ipportalegre.pt
* Correspondence: acsf@fc.ul.pt; Tel.: +35-12-1750-0257

plants

MDPI

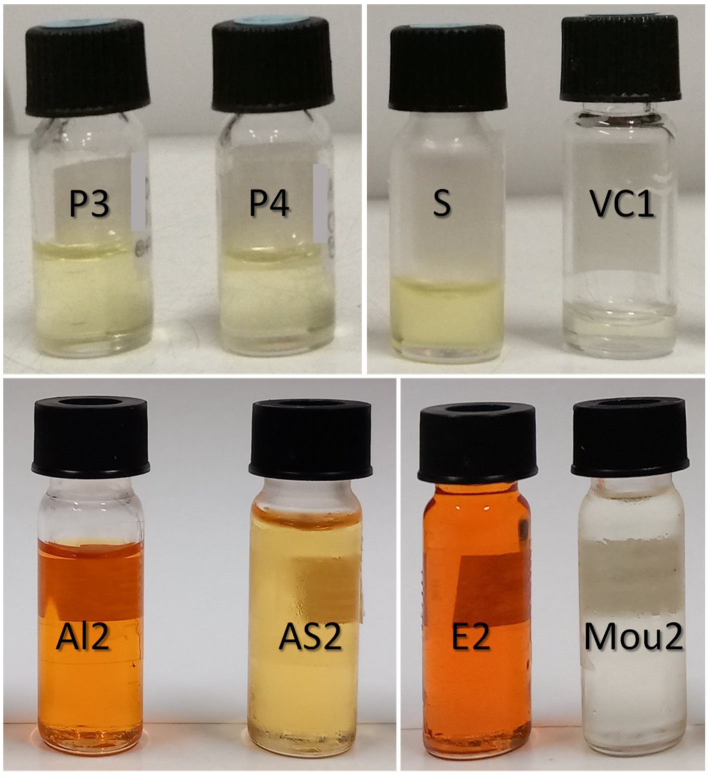

Figure S1. Detail of the essential oils' colours obtained from several oregano accessions. Portalegre (P3 and P4), Santarém (S), Viana do Castelo (VC1), Alandroal (Al2), Alcácer do Sal (AS2), Elvas (E2), Moura (Mou2). For harvest site codes, *vide* Table 1.

plants

MDPI

Table S1a. Percentage composition of the EOs isolated by hydrodistillation, from *Origanum vulgare* subsp. *virens* accessions obtained from Alandroal, Alcácer do Sal, Alter do Chão, Arronches, Bragança, Castelo Branco, Elvas, Estremoz, Grândola, Guarda, in 2021 and 2022.

**Components**	**RI**	**Accessions**																	
		**Al***		**AS***		**AC***		**Ar***		**B†**		**CB†**	**E***		**Es***		**Gr***		**G†**
		**2021**	**2022**	**2021**	**2022**	**2021**	**2022**	**2021**	**2022**	**2022**	**2022**	**2022**	**2021**	**2022**	**2021**	**2022**	**2021**	**2022**	**2022**
Tricyclene	921	t	t		t		t		t			t	t	t	t	t	t	t	t
α-Thujene	924	3.7	3.4	3.4	3.0	0.2	2.6	0.2	0.4	0.6	0.5	1.2	1.7	3.5	1.9	2.9	2.1	2.1	1.0
Benzaldehyde	927									t	t								
α-Pinene	930	1.5	1.4	1.4	1.3	0.1	1.0	0.1	0.3	0.4	0.4	0.5	0.8	1.5	0.9	1.2	0.9	0.8	0.4
Camphene	938	0.2	0.2	0.2	0.1	0.1	0.2	t	t	0.1	0.1	0.1	0.1	0.2	0.3	0.4	0.2	0.3	0.1
Sabinene	958	0.1	0.2	0.1	0.2	0.2	0.2		0.1	2.4	4.1	0.6		0.1	0.1	0.2	0.1	0.2	0.3
1-Octen-3-ol	961							0.4	0.1				0.1						
3-Octanone	961	0.1	0.1	0.1	0.1	t	0.1	0.2	0.1	1.0	1.4	0.2	t	0.1	t	0.1	t	0.1	0.1
β-Pinene	963	0.8	0.7	0.8	0.4	0.1	0.6	0.2	0.9	0.6	0.8	0.5	0.6	0.7	0.5	0.6	0.5	0.6	0.5
Dehydro-1,8-cineole *	973	t		t	t	0.2	0.1	t	t	t	t		t	t	t	t	t	t	t
3-Octanol	974		t				t	t	0.2	0.6	0.5	0.7							0.5
β-Myrcene	975	3.3	2.7	3.5	2.5	0.3	2.0	0.5	0.4	1.4	1.2	1.5	1.9	2.3	2.1	2.3	2.1	2.3	1.2
α-Phellandrene	995	0.5	0.4	0.5	0.4		0.3	t	0.1	0.2	0.1	0.3	0.2	0.3	0.3	0.3	0.3	0.3	0.2
δ-3-Carene	1000	0.2	0.1	0.2	0.1		0.1		0.1				0.1	0.2	0.1	0.1	0.1	0.1	
Benzene acetaldehyde	1002									t	t	0.1							t
α-Terpinene	1002	4.9	3.6	5.8	4.7	0.3	2.7	0.2	0.3	2.7	1.3	3.9	2.3	2.8	2.3	2.4	3.5	2.8	2.3
p-Cymene	1003	**27.1**	**17.6**	**24.5**	**22.5**	5.3	19.0	0.3	1.0	1.8	0.8	4.6	15.9	23.1	7.5	11.5	19.4	10.1	7.4
β-Phellandrene	1005	0.5	0.4	0.4	0.3	0.1	0.3	t	0.1	0.6	0.8	0.2	0.3	0.4	0.2	0.3	0.3	0.2	0.2
Limonene	1009	0.7	0.5	0.7	0.5	0.2	0.7	t	0.1	1.0	0.6	0.4	0.3	0.5	0.3	0.5	0.5	0.4	0.3
cis-β-Ocimene	1017	1.7	0.9	1.5	0.8	0.5	1.2	4.0	2.8	**10.8**	**13.0**	5.5	0.5	0.5	1.6	0.9	2.7	3.7	4.2
trans-β-Ocimene	1027	0.3	0.2	0.3	0.2	0.2	0.2	0.5	0.3	**8.8**	**13.4**	4.1	0.1	0.1	0.3	0.2	0.3	0.4	3.6
γ-Terpinene	1035	**21.2**	14.0	**32.1**	**26.8**	2.4	11.7	0.7	1.7	**13.4**	**4.4**	**40.0**	12.4	11.4	12.2	12.2	17.1	19.5	**35.0**

**Components**	**RI**	**Accessions**																	
		**Al***		**AS***		**AC***		**Ar***		**B†**		**CB†**	**E***		**Es***		**Gr***		**G†**
		**2021**	**2022**	**2021**	**2022**	**2021**	**2022**	**2021**	**2022**	**2022**	**2022**	**2022**	**2021**	**2022**	**2021**	**2022**	**2021**	**2022**	**2022**
trans-Sabinene hydrate	1037	0.7	0.5	0.7	0.3	0.1	0.4	0.1	0.1		t	0.1	0.4	0.4	0.2	0.3	0.4	0.4	
cis-Linalool oxide (furanoid)	1045						t	0.3	0.2	t		0.1			0.1		t	t	0.2
2,5-Dimethyl styrene	1059	t	t	t	t	0.1	t			0.1	0.3		t	0.1			t		
trans-Linalool oxide (furanoid)	1059							0.5	0.6						0.1	0.2		0.2	t
Terpinolene	1064	0.2	0.1	0.2	0.1	0.1	0.1	0.1	0.1	0.6	0.1	0.1	0.2	0.2	0.1	0.1	0.1	0.1	0.1
cis-Sabinene hydrate	1066	0.2	0.2	0.2	0.1	0.1	0.1		0.1			t	0.1	0.1	0.1	0.1		0.1	t
Linalool	1074	0.5	0.3	0.2	0.2	0.8	1.4	**81.5**	**80.4**	2.7	2.5	2.9	0.3	1.2	17.3	19.9	0.4	17.2	17.7
trans-*p*-2-Menthen-1-ol	1099	0.1	t		t		t		t					t		t		t	
Cosmene *	1102					0.1	t	t											
allo-Ocimene	1110	0.1	t	t	t		t	0.1	0.1	0.4	0.3	t	t	t	t	t	0.1	t	t
trans-Limonene oxide	1112						t	t	t									t	
1,3,8-*p*-Menthatriene	1114		t		t		t		t					t		t			
cis-Linalool oxide (pyranoid)	1132								t										
Borneol	1134	0.3	0.2	0.1	0.1	0.1	0.2	0.1	t	t	t	0.1	0.2	0.1	0.5	0.3	0.2	0.3	0.2
trans-Linalool oxide (pyranoid)	1143								0.1									t	
Terpinen-4-ol	1148	1.2	0.9	1.1	0.8	1.1	0.7	0.2	0.3	1.9	1.0	0.8	1.1	0.9	0.7	0.7	0.7	0.5	0.5
Methyl salicylate	1159				t		0.1			0.2	0.1	t		t		t		t	t
α-Terpineol	1159	0.2	0.1	0.2	0.1	8.1	8.2	0.1	0.1	0.3		0.1	0.1	0.1	0.1	0.2	0.1	0.2	0.1
cis-Dihydrocarvone	1159								t				t	0.1	t	t		t	
Myrtenol	1168	0.1																	
Cuminaldehyde	1200		t		t		t							0.1		t			t
Nerol	1206				1.7			t	t	t									
Pulegone	1207				2.9						1.7								
Carvone	1210	t					0.3						0.2	0.1	0.1		0.2		
Thymoquinone	1210	0.4											0.3	0.1					

Components	RI	Accessions																	
		**Al***		**AS***		**AC***		**Ar***		**B†**		**CB†**	**E***		**Es***		**Gr***		**G†**
		**2021**	**2022**	**2021**	**2022**	**2021**	**2022**	**2021**	**2022**	**2022**	**2022**	**2022**	**2021**	**2022**	**2021**	**2022**	**2021**	**2022**	**2022**
Thymol methyl ether	1210	2.1	1.4	2.2		0.5	1.2		t	0.5		1.2			0.3	0.4	0.4	t	2.4
Carvacrol methyl ether	1224	5.9	4.6	4.8		3.7	4.7	0.8	1.7	0.8		1.3	4.9	5.4	3.0	3.6	1.0	1.1	0.8
Geraniol	1236							t	0.1	0.7						t		t	t
p-Cymen-7-ol	1265	0.1	t	t	t	t		t				t	t	t	t	t	0.1	t	t
Thymol	1275	15.3	**40.7**	10.9	**27.2**	**50.2**	**34.0**	0.1	0.4	2.7		17.3	0.4	0.3	16.1	11.4	**39.4**	0.7	8.9
Carvacrol	1286	0.4	0.3	0.2	0.1	10.4	1.5	2.3	1.4			0.1	**51.5**	**40.4**	**27.7**	**23.1**	2.7	**25.3**	t
Thymol acetate	1327		t	t		t	t						t	t	t				
Eugenol	1327								t	0.1	t	t						t	t
δ-Elemene	1332		t		t		t	t	t	0.2	0.2	0.1		t		t		t	0.1
α-Cubebene	1345	t	t	t	t	0.1	t							t	t	t	t		
Geranyl acetate	1370									0.2									
α-Ylangene	1371	t	t		t		t						t	t		t		t	
α-Copaene	1375	0.1	0.1	0.1	t	0.4	t	t	t	t	t	t	t	t	t	t	0.1	t	0.1
β-Bourbonene	1379	t	t	t	t	0.1	0.1	t	t	0.8	0.6	0.1	t	t	t	t	t	t	0.1
β-Cubebene	1385		t		t		t		t							t		t	
β-Elemene	1388		t		t		t	t	t	0.2	0.1	0.1		t		t	t	t	t
β-Caryophyllene	1414	1.5	1.0	0.8	0.4	2.0	1.2	3.0	2.9	**16.6**	**18.5**	4.1	0.8	1.0	1.2	1.2	0.9	3.5	4.4
β-Copaene *	1426	0.1	0.1	0.1	t	0.1	t	t	t	0.2	0.2	t	t	t	t	t	0.1	t	t
Aromadendrene	1428	0.2	0.2				0.1		t			t	0.1	0.1		t	0.2	0.1	
trans-α-Bergamotene	1434									t	0.1	t							t
β-Ylangene	1435									0.1	t								
α-Humulene	1447	0.3	0.2	0.1	0.1	0.3	0.2	0.5	0.5	2.0	2.5	0.6	0.1	0.1	0.2	0.2	0.1	0.6	0.8
allo-Aromadendrene	1456		t			t			t						t				t
γ-Muurolene	1469	0.2	0.2	0.2	0.1	0.4	t	t	0.1		t	t	0.1	0.1	0.2	t	0.2	0.1	t
Germacrene D	1474	0.4	0.6	0.2	0.3	0.6	0.8	1.2	0.9	8.4	7.6	3.0	0.2	0.3	0.3	0.7	0.3	1.3	1.9

Components	RI	Accessions																	
		**Al***		**AS***		**AC***		**Ar***		**B†**		**CB†**	**E***		**Es***		**Gr***		**G†**
		**2021**	**2022**	**2021**	**2022**	**2021**	**2022**	**2021**	**2022**	**2022**	**2022**	**2022**	**2021**	**2022**	**2021**	**2022**	**2021**	**2022**	**2022**
β-Selinene	1476	t	t											t		t	t		
Valencene	1484		t		t		t							t		t			t
Bicyclogermacrene	1487	0.6	0.6	0.2	0.1	0.4	0.5	0.9	0.6	1.4	1.7	0.9		0.2	0.2	0.1	0.3	0.9	1.7
Viridiflorene	1487												0.1						
α-Muurolene	1494	0.1	0.1	t	t	0.1	t	t	t	0.2	0.2	t	t	t	t	t	t	t	t
β-Bisabolene	1500	0.6		1.2		4.7		0.3					0.7		0.6		0.8		
trans-Calamenene	1505	t	t		t		t						t	t	t		t		
δ-Cadinene	1505	0.3	0.2	0.2	0.1	0.7	0.2	0.1	0.1	0.4	0.3	0.2	0.2	0.1	0.2	0.1	0.3	0.1	0.1
α-Calacorene	1525		t		t		t							t					
Cadina-1,4-diene	1529	t				0.1									t				
α-Cadinene	1529	t	t	t	t	t	t		t					t	t	t	t	0.1	t
β-Caryophyllene oxide	1561		0.1	0.1	t	0.3	0.2	0.3	0.2	1.0	1.7	0.1	0.1	0.1	0.1	0.1	0.2	0.2	0.3
Globulol	1566	t	t		t		t	t	t					t		t		t	
Humulene epoxide *	1580							t											
T-Cadinol	1616		t		t		t		t	0.1	0.1	t		t		t		t	t
α-Cadinol	1616	0.1	0.1		t	0.1	t	0.1	t	0.4	0.4	0.1	t	0.1	t	0.1	t	t	0.3
α-Muurolol	1618	t		t				t	t										
α-Eudesmol	1634			t		0.2							0.1		0.2		0.2		
α-Bisabolol	1656			t	t	t												t	
**% Identification**		99.1	99.2	99.5	98.6	96.2	99.2	99.9	100.0	89.6	90.4	97.8	99.5	99.4	100.0	99.0	99.6	96.9	98.0
Monoterpene hydrocarbons		67.0	46.4	75.6	63.9	10.2	42.9	6.9	8.8	45.8	41.9	63.5	37.4	47.8	30.7	36.1	50.3	43.9	56.8
Oxygen-containing monoterpenes		27.5	49.2	20.6	33.5	75.3	52.8	86.0	85.5	9.8	12.0	24.0	59.5	49.3	66.2	60.3	45.6	46.0	30.8
Sesquiterpene hydrocarbons		4.4	3.3	3.1	1.1	10.0	3.1	6.0	5.1	30.5	32.0	9.1	2.3	1.9	2.9	2.3	3.3	6.7	9.2

**Components**	**RI**	**Accessions**																	
		**Al***		**AS***		**AC***		**Ar***		**B†**		**CB†**	**E***		**Es***		**Gr***		**G†**
		**2021**	**2022**	**2021**	**2022**	**2021**	**2022**	**2021**	**2022**	**2022**	**2022**	**2022**	**2021**	**2022**	**2021**	**2022**	**2021**	**2022**	**2022**
Oxygen-containing sesquiterpenes		0.1	0.2	0.1	t	0.6	0.2	0.4	0.2	1.5	2.2	0.2	0.2	0.2	0.3	0.2	0.4	0.2	0.6
Others		0.1	0.1	0.1	0.1	0.1	0.2	0.6	0.4	2.0	2.3	1.0	0.1	0.2	t	0.1	t	0.1	0.6

RI: In-lab calculated retention index relative to C_9-C_{17} *n*-alkanes on the DB-1 column. t: traces (<0.05%). Al: Alandroal, AS: Alcácer do Sal. AC: Alter do Chão. Ar: Arronches. B: Bragança. CB: Castelo Branco. E: Elvas. Es: Estremoz. Gr: Grândola. G: Guarda. * rooted cuttings † nutlets. Bold: dominant compounds in each accession.

Table S1b. Percentage composition of the EOs isolated by hydrodistillation, from *Origanum vulgare* subsp. *virens* accessions obtained from Marvão, Mora, Moura, Nisa, Portalegre, Redondo, Santarém, Serpa, Sousel, Viana do Castelo, in 2021 and 2022.

**Components**	**RI**	**Accessions**																			
		**M***		**Mo***		**Mou***		**N***	**P***		**P†**		**R***		**S†**	**Se***		**So***		**VC†**	
		**2021**	**2022**	**2021**	**2022**	**2021**	**2022**	**2022**	**2021**	**2022**	**2022**	**2022**	**2021**	**2022**	**2022**	**2021**	**2022**	**2021**	**2022**	**2022**	**2022**
Tricyclene	921	t	t	t	t	t	t	t	t	t	t	t	t	t	t	t	t	t	t	t	t
α-Thujene	924	1.2	2.2	2.3	3.3	0.2	0.8	2.1	t	0.7	1.3	1.8	1.6	2.8	1.7	0.6	1.5	3.3	5.0	0.1	1.4
Benzaldehyde	927										t	t								0.1	
α-Pinene	930	0.4	0.7	0.8	1.4	0.1	0.3	0.8	t	0.3	0.6	0.8	0.7	1.2	0.6	0.2	0.6	1.1	1.6	t	0.6
Camphene	938	0.2	0.2	0.3	0.2	t	0.1	0.2	t	0.2	0.2	0.2	0.2	0.2	0.1	0.1	0.1	0.1	0.2	t	0.2
Sabinene	958	0.1	0.3	0.2	0.3		t	0.2		0.8	0.4	0.3	0.1	0.2	0.6		t	0.3	0.4	0.8	1.1
1-Octen-3-ol	961					0.4	0.1		0.3							0.1	0.1				
3-Octanone	961	0.1	0.1	0.1	0.1	0.2	0.1	0.1	0.2	0.3	0.1	0.1	0.1	0.1	0.2	t	0.1	0.1	0.1	0.6	0.4
β-Pinene	963	0.8	0.8	0.6	0.6	0.2	1.1	0.5		0.6	0.5	0.4	0.6	0.6	0.8	0.5	1.0	0.5	0.8		
Dehydro-1,8-cineole *	973		t	t	t	t	t	t		t	0.2	t	t	t	t	t	t	t	t	t	t
3-Octanol	974					0.1	t			0.2	0.6	0.7			0.4	0.1	0.4		t	0.5	0.8
β-Myrcene	975	1.8	2.6	1.9	2.3	0.3	0.9	2.1	0.1	0.8	1.5	1.8	1.8	2.4	1.9	0.5	1.4	2.1	2.4	1.2	2.0
α-Phellandrene	995	0.2	0.3	0.3	0.4		0.1	0.3		0.1	0.2	0.4	0.2	0.4	0.3	0.1	0.2	0.4	0.5	0.1	0.3
δ-3-Carene	1000	0.1	0.1	0.1	0.1		0.1	0.1		t			0.1	0.1	t	t	0.1	0.1	0.1		
Benzene acetaldehyde	1002										0.1	0.1			t					t	0.1
α-Terpinene	1002	1.9	2.5	3.1	3.9	0.2	0.7	4.9	0.1	0.9	3.0	4.5	2.5	3.8	3.9	0.7	2.4	3.7	4.6	1.0	3.0
p-Cymene	1003	9.7	15.1	19.1	23.6	0.8	3.1	14.6	0.2	2.8	9.4	7.4	13.8	19.3	4.7	3.9	9.0	12.7	15.6	2.0	9.4
β-Phellandrene	1005	0.2	0.3	0.3	0.3	t	0.1	0.3		0.1	0.2	0.2	0.2	0.3	0.2	0.1	0.2	0.4	0.6	0.2	0.2
Limonene	1009	0.3	0.6	0.4	0.5	t	0.3	0.4	t	0.3	0.6	0.4	0.3	0.6	0.4	0.1	0.4	0.4	0.5	0.4	0.4
cis-β-Ocimene	1017	3.4	2.6	2.8	2.2	0.8	0.9	1.4	2.0	2.6	2.0	2.4	3.7	1.9	4.2	0.9	1.6	1.6	1.4	10.2	10.2
trans-β-Ocimene	1027	0.4	0.3	0.3	0.2	0.1	0.2	0.6	0.3	2.6	0.6	0.5	0.5	0.2	1.9	0.2	0.3	0.3	0.2	6.5	5.8
γ-Terpinene	1035	12.3	13.8	17.2	18.7	0.5	2.3	24.8	0.4	5.7	**27.3**	**36.9**	18.6	15.9	**31.5**	2.7	14.5	12.6	12.5	7.7	**39.3**
trans-Sabinene hydrate	1037	0.3	0.4	0.4	0.5	0.1	0.1	0.5	t	0.3			0.4	0.4	0.1	0.1	0.2	0.6	1.0		

**Components**	**RI**	**Accessions**																			
		**M***		**Mo***		**Mou***		**N***	**P***		**P†**		**R***		**S†**	**Se***		**So***		**VC†**	
		**2021**	**2022**	**2021**	**2022**	**2021**	**2022**	**2022**	**2021**	**2022**	**2022**	**2022**	**2021**	**2022**	**2022**	**2021**	**2022**	**2021**	**2022**	**2022**	**2022**
cis-Linalool oxide (furanoid)	1045	t	t	t	t	0.1	0.2		t	0.1	0.1	t	t	t	t	0.1	0.1			t	
2,5-Dimethyl styrene	1059							t		t	t	t		t				t	t	t	t
trans-Linalool oxide (furanoid)	1059	0.3	0.2	0.2	0.1	0.3	0.4		0.2	0.4			0.2			0.2	0.1				
Terpinolene	1064	0.1	0.1	0.1	0.1	t	0.1	0.1	t	0.1	0.1	0.1	0.1	0.1	0.1	t	0.1	0.5	0.7	0.2	0.1
cis-Sabinene hydrate	1066	0.1	0.1	0.1	0.2	t		0.2		0.1	0.1	0.1	0.1	0.1	0.1		0.1	0.9	1.5		t
Linalool	1074	**26.0**	**25.3**	11.0	7.4	**84.1**	**69.9**	0.3	**80.5**	**58.0**	0.3	0.2	15.6	0.4	5.9	**70.4**	**32.9**	0.2	0.5	**20.3**	1.6
trans-*p*-2-Menthen-1-ol	1099		t		t			t		t				t			t		0.2		
Cosmene *	1102	t				t			t							t			t	0.1	
allo-Ocimene	1110	t	t	0.1	0.1	t	t	t	t	t	0.1	t	0.1	t	0.1	t	t	0.1	0.1	0.2	0.1
trans-Limonene oxide	1112																				
1,3,8-*p*-Menthatriene	1114		t		t		t			t				t			t		t		
cis-Linalool oxide (pyranoid)	1132					t	0.1									t					
Borneol	1134	0.4	0.2	0.4	0.2	0.1	0.1	0.2	0.2	0.3	0.2	0.2	0.4	0.2	0.2	0.2	0.1	0.2	0.1	0.1	0.2
trans-Linalool oxide (pyranoid)	1143		t				t			t							t				
Terpinen-4-ol	1148	0.6	0.6	0.7	0.7	0.1	0.3	0.6	0.2	0.4	0.8	0.9	0.5	0.9	0.8	0.3	0.4	4.0	4.3	1.3	0.7
Methyl salicylate	1159				t		t	t		t	0.1	t		t					t	0.1	t
α-Terpineol	1159	0.2	0.1	0.1	0.1	0.1	0.1	0.1	0.1	0.1	17.6	1.9	0.1	0.1	1.1	0.1	0.1	0.4	0.4	0.2	0.1
cis-Dihydrocarvone	1159		t							t											
Myrtenol	1168													t							
Cuminaldehyde	1200		t		t			t		t	t	t		t			t		t	t	t
Nerol	1206					0.1	t									0.1				t	
Pulegone	1207																				
Carvone	1210		0.1	0.2									t								
Thymoquinone	1210																				
Thymol methyl ether	1210	2.8	3.7	2.1	2.1	0.3	0.6	1.8	t	0.2	3.3	2.7		0.5	0.5	0.9	2.2	0.1	0.1	0.2	3.8

**Components**	**RI**	**Accessions**																			
		**M***		**Mo***		**Mou***		**N***	**P***		**P†**		**R***		**S†**	**Se***		**So***		**VC†**	
		**2021**	**2022**	**2021**	**2022**	**2021**	**2022**	**2022**	**2021**	**2022**	**2022**	**2022**	**2021**	**2022**	**2022**	**2021**	**2022**	**2021**	**2022**	**2022**	**2022**
Carvacrol methyl ether	1224	4.1	4.1	2.8	2.7	0.3	0.8	2.9	2.6	1.6	1.3	2.1	2.1	1.8	1.6	1.1	1.8	0.1	0.1	4.6	1.1
Geraniol	1236		t			0.1	0.1		t	t						0.1	0.1			t	
p-Cymen-7-ol	1265	t	t	0.1	t		t	t		t	t	t	t	t	t	t	t	t	t	0.1	0.1
Thymol	1275	15.7	11.0	**24.6**	**21.2**	4.4	10.5	**34.3**	1.0	3.9	16.6	27.2	0.4	**40.4**	24.5	11.1	23.0	**43.3**	**38.7**	1.2	8.0
Carvacrol	1286	9.2	6.3	0.2	0.2	0.4	0.5	0.4	0.3	5.6	0.2	0.1	**24.8**	1.2	1.0	1.4	0.2	6.6	2.3	0.5	t
Thymol acetate	1327		t				t										t	t	t		
Eugenol	1327														t					t	t
δ-Elemene	1332		t		t		t	t		0.1	0.1	0.1		t	0.2		t		t	0.4	0.1
α-Cubebene	1345			t	t			t					t	0.1		t	t	t	t		
Geranyl acetate	1370																				
α-Ylangene	1371		t		t		t	t		t				t					t		
α-Copaene	1375		t	t	t	t	t	t	t	t	t	t	t	t	t	t	t	0.1	t	t	t
β-Bourbonene	1379	0.1	0.1	0.1	t	0.1	t	t	0.2	0.1	0.1	t	t	t	t	0.1	0.1	t	t	0.8	0.1
β-Cubebene	1385																t		t		
β-Elemene	1388		t	t	t	t	0.1	t	t	0.1	t	t	t	t	t	t	t		t	0.2	0.1
β-Caryophyllene	1414	2.5	2.0	1.8	1.5	1.6	2.0	1.6	3.7	3.6	1.6	1.7	3.2	0.8	3.9	1.1	1.6	0.7	0.7	11.5	2.1
β-Copaene *	1426		t		t	t	t	t	t	t	t	t	t	0.1	t	t	t	t	t	0.2	t
Aromadendrene	1428		t	0.1	t		t	t		t	t	t		0.1	t	t	t				t
trans-α-Bergamotene	1434										0.1	t					t		0.1	0.1	t
β-Ylangene	1435																		t		
α-Humulene	1447	0.4	0.3	0.3	0.2	0.3	0.3	0.3	0.5	0.6	0.3	0.3	0.5	t	0.7	0.2	0.3	0.1	0.1	1.1	0.3
allo-Aromadendrene	1456			t	t				t	t				t	t	t				0.1	t
γ-Muurolene	1469	t	0.1	0.7	0.5	0.4	0.2	0.1	t	t	t	t	0.5	0.1	t	t	0.3	0.1	0.1	t	t
Germacrene D	1474	1.8	1.5	1.0	1.2	1.5	1.1	0.6	3.0	2.1	0.8	1.0	0.8	0.7	1.5	1.2	0.5	0.2	0.5	7.6	0.9
β-Selinene	1476			t	t			t		t				t					t		

**Components**	**RI**	**Accessions**																			
		**M***		**Mo***		**Mou***		**N***	**P***		**P**†		**R***		**S**†	**Se***		**So***		**VC**†	
		**2021**	**2022**	**2021**	**2022**	**2021**	**2022**	**2022**	**2021**	**2022**	**2022**	**2022**	**2021**	**2022**	**2022**	**2021**	**2022**	**2021**	**2022**	**2022**	**2022**
Valencene	1484		t		t		t	t		t				t							
Bicyclogermacrene	1487	0.6	0.4	0.7	0.6	0.4	0.4	0.4	1.6	1.3	1.1	0.8	0.9	0.4	1.8	0.3	0.3	0.1	0.1	5.5	2.5
Viridiflorene	1487																				
α-Muurolene	1494		t	0.1	t	t	t	t		t	t	t	t	0.1	t	t	t	t	t	t	t
β-Bisabolene	1500	1.0		1.3		1.0			2.3				3.2			0.5		1.0			
trans-Calamenene	1505													t				0.3	t		
δ-Cadinene	1505	0.2	0.1	0.3	0.2	t	0.1	0.1	t	0.1	0.1	0.1	0.1	0.2	0.2	0.1	0.1	0.2	0.2	0.4	0.1
α-Calacorene	1525				t			t						t					t		
Cadina-1,4-diene	1529			t												t				t	
α-Cadinene	1529		t	t	t		t	t		t			t	t		0.1	t	t	t	t	
β-Caryophyllene oxide	1561	0.3	0.1	0.3	0.2	0.1	0.1	0.2	0.3	0.3	0.2	0.1	0.2	0.1	0.1	0.1	0.2	0.1	0.1	1.0	0.1
Globulol	1566		t		t		t	t		t				t			t		t		
Humulene epoxide *	1580																				
T-Cadinol	1616		t		t		t	t		t	t	t		t	0.1		t		t	0.4	0.1
α-Cadinol	1616		t	t	0.1	t	t	0.1	t	0.1	0.1	0.1	t	0.1	0.2	t	t		t	0.4	0.1
α-Muurolol	1618														t						
α-Eudesmol	1634	0.1		0.1												t		t			
α-Bisabolol	1656			t				t			t	t	0.1		t				t		
**% Identification**		99.9	99.3	99.7	98.2	99.8	99.3	98.2	100.0	98.5	94.1	98.6	99.3	98.9	98.1	100.0	98.7	99.6	98.4	90.2	97.5
Monoterpene hydrocarbons		33.1	42.5	49.9	58.2	3.2	11.1	53.4	3.1	18.6	48.0	58.1	45.1	50.0	53.0	10.6	33.4	40.2	47.2	30.7	74.1
Oxygen-containing monoterpenes		59.7	52.1	42.9	35.4	90.5	83.7	41.3	85.1	71.0	40.7	35.4	44.6	46.0	35.8	86.1	61.3	56.4	49.2	28.5	15.6
Sesquiterpene hydrocarbons		6.6	4.5	6.4	4.2	5.3	4.2	3.1	11.3	8.0	4.2	4.0	9.2	2.6	8.3	3.6	3.2	2.8	1.8	27.9	6.2
Oxygen-containing sesquiterpenes		0.4	0.1	0.4	0.3	0.1	0.1	0.3	0.3	0.4	0.3	0.2	0.3	0.2	0.4	0.1	0.2	0.1	0.1	1.8	0.3

**Components**	**RI**	**Accessions**																			
		**M***		**Mo***		**Mou***		**N***	**P***		**P†**		**R***		**S†**	**Se***		**So***		**VC†**	
		**2021**	**2022**	**2021**	**2022**	**2021**	**2022**	**2022**	**2021**	**2022**	**2022**	**2022**	**2021**	**2022**	**2022**	**2021**	**2022**	**2021**	**2022**	**2022**	**2022**
Others		0.1	0.1	0.1	0.1	0.7	0.2	0.1	0.5	0.5	0.9	0.9	0.1	0.1	0.6	0.2	0.6	0.1	0.1	1.3	1.3

RI: In-lab calculated retention index relative to C_9-C_{17} *n*-alkanes on the DB-1 column. t: traces (<0.05%). M: Marvão. Mo: Mora. Mou: Moura. N: Nisa. P: Portalegre. R: Redondo. S: Santarém. Se: Serpa. So: Sousel. VC: Viana do Castelo. * rooted cuttings. † nutlets. Bold: dominant compounds in each accession.

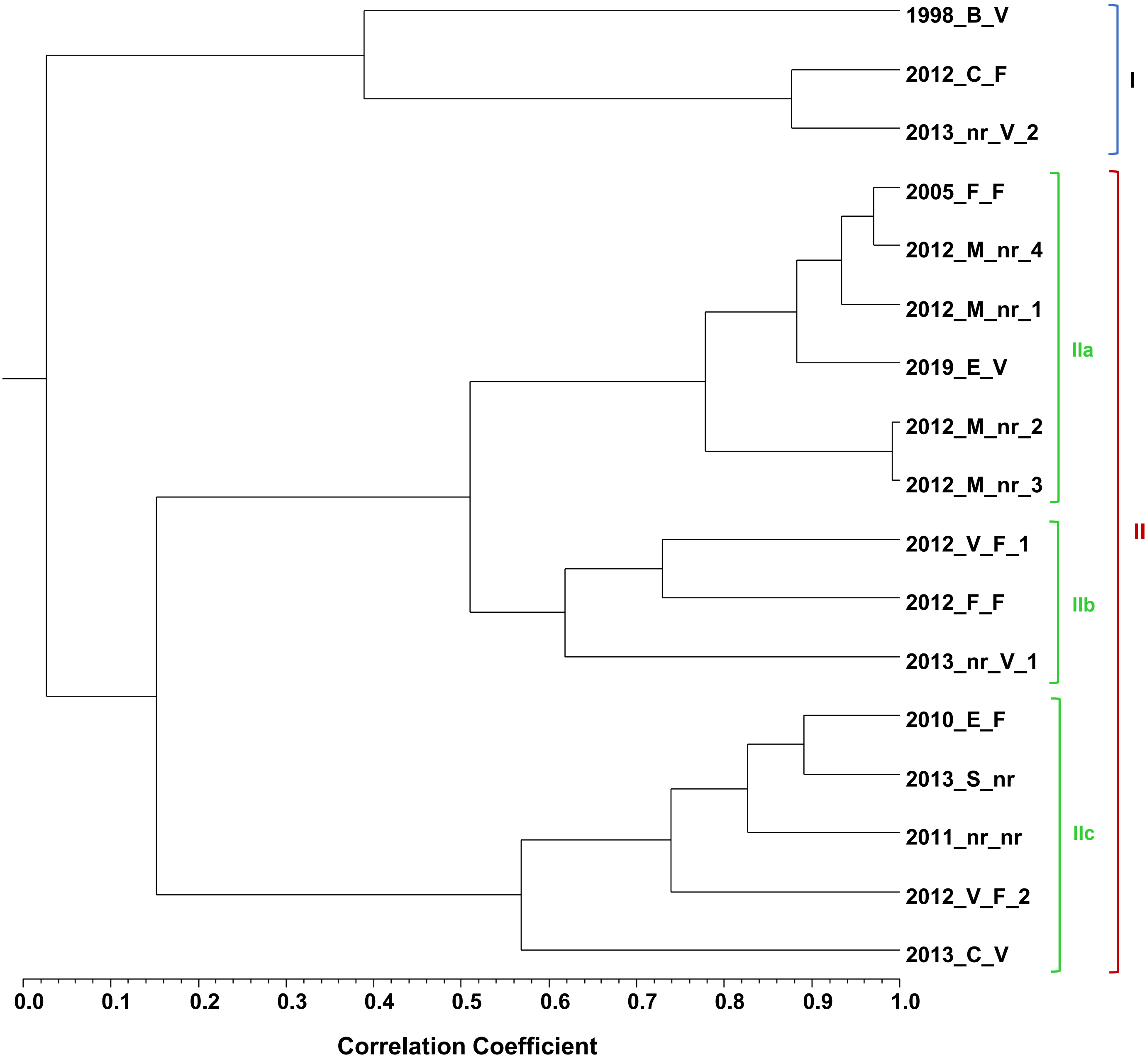

Figure S2. Dendrogram obtained by cluster analysis of the reported percentage composition of the essential oils isolated by hydrodistillation from Portuguese *Origanum vulgare*, based on correlation, and using the unweighted pair-group method with arithmetic average (UPGMA). In the code, the year is followed by the first letter of the district of harvest and the second letter refers to the plant stage. For harvest site codes, *vide* Table 3.
